# Radiocapitellar joint pressures following transradial amputation increase during elbow motion

**DOI:** 10.1038/s41598-021-92743-6

**Published:** 2021-07-06

**Authors:** Young-Hoon Jo, Bong-Gun Lee, Chang-Hun Lee, Kwang-Hyun Lee, Dong-Hong Kim, Doo-Sup Kim, Sung Jae Kim

**Affiliations:** 1 Department of Orthopedic Surgery, Hanyang University Guri Hospital, Guri, South Korea; 2 Department of Orthopedic Surgery, Hanyang University Seoul Hospital, Seoul, South Korea; 3 Department of Orthopedic Surgery, Wonju College of Medicine, Yonsei University, Wonju Severance Christian Hospital, Wonju, South Korea; 4 Department of Orthopaedic Surgery, Dongtan Sacred Heart Hospital, Hallym University College of Medicine, Hwaseong, Republic of Korea

**Keywords:** Health care, Medical research, Rheumatology

## Abstract

This study aimed to compare the contact area, mean pressure, and peak pressure of the radiocapitellar joint (RCJ) in the upper limb after transradial amputation with those of the normal upper limb during elbow flexion and forearm rotation. Testing was performed using ten fresh-frozen upper limbs, and the transradial amputation was performed 5 cm proximal to the radial styloid process. The specimens were connected to a custom-designed apparatus for testing. A pressure sensor was inserted into the RCJ. The biomechanical indices of the RCJ were measured during elbow flexion and forearm rotation in all specimens. There was no significant difference in the contact area between the normal and transradial amputated upper limbs. However, in the upper limbs after transradial amputation, the mean pressure was higher than that in the normal upper limbs at all positions of elbow flexion and forearm rotation. The peak pressure was significantly higher in the upper limbs after transradial amputation than in the normal upper limbs, and was especially increased during pronation at 45° of elbow flexion. In conclusion, these results could cause cartilage erosion in the RCJ of transradial amputees. Thus, methods to reduce the pressure of the RCJ should be considered when a myoelectric prosthesis is developed.

## Introduction

Upper limb amputations cause greater disability in activities of daily living than lower limb amputations^1^. Such amputations are mostly caused by trauma, or to a smaller extent, are secondary to malignant neoplasm or infection^2^. The number of major upper extremity amputees was estimated to be 41,000 in the United States in 2005^3^. Transradial amputations account for approximately half of major upper limb amputations^2,4^, and the use of a prosthesis was reported to be the highest in transradial amputees^5^. With the development of bioengineering, myoelectric prostheses have gradually been designed to mimic human hands and are more practical^6,7^. For amputees, training and the proper use of a prosthesis improves functional outcomes and make it possible to more rapidly return to work and daily living^8^.

One of the limitations of the myoelectric prosthesis is the restoration of active forearm rotation^9^. Pronation and supination of the forearm are frequently performed during activities of daily living, including driving a car, opening and closing a door, and using a spoon. The most common suspension method of the myoelectric prosthesis is the self-attachable and removable socket, which is designed to conform over the residual limb. However, the socket may interfere with the rotation of the forearm by mechanically blocking this motion in transradial amputees^10,11^. When forearm rotation is lost, the patient alters his or her upper arm and torso movements to compensate^12,13^. Such compensatory movements often result in residual limb pain, secondary musculoskeletal problems, and overuse syndromes over time^14^. While the socket suspension method interferes with the rotation of the forearm, osseointegration, which anchors a prosthesis directly to the skeleton, can restore natural forearm rotation successfully in transradial amputees^10^. In addition, a longer residual limb allows better active forearm rotation^15^.

The structures involved in the rotation of the forearm are the proximal radioulnar joint, interosseous membrane, and the distal radioulnar joint (DRUJ). These structures have been shown to function as an integrated osseoligamentous system to distribute the applied load^16^. A normal DRUJ is lost in transradial amputees. In addition, the distal oblique bundle (DOB), which makes an important contribution to the stability between the radius and ulna, is also lost in these patients^17^. The disruption of these stabilisers can lead to forearm axial instability^18^. In these patients, biomechanical changes may occur in the radiocapitellar joint (RCJ) of the elbow, though this topic is not well known. If biomechanical changes occur, these factors should be considered in the development of myoelectric prostheses.

The purpose of this study was to investigate changes in contact area, mean pressure, and peak pressure in the RCJ at various elbow flexion and forearm rotation positions in normal upper limbs and in upper limbs after transradial amputation. We hypothesise that the contact area and pressure of the RCJ increases following transradial amputation, and that the differences in biomechanical indices are dependent on elbow flexion and forearm rotation positions.

## Methods

### Specimen preparation

All experimental protocols were approved by Institutional Review Board of the Wonju Severance Christian Hospital (CR32011) and all methods were carried out in accordance with relevant guidelines and regulations. The informed consent was waived by the ethical committee which approved the study. Ten fresh-frozen upper limbs were obtained from Wonju Severance Christian Hospital. The average age was 72.3 years (58–83 years), and there were six male and four female donors. The specimens were stored at − 20 °C until thawed at room temperature for 24 h prior to the experiment. The specimens were examined to ensure that none had a flexion contracture of more than 10° and a pronosupination rotation arc of less than 140°. In addition, C-arm fluoroscopy was used to detect radiologic evidence of arthritis in the elbow joint. All specimens demonstrated a normal passive range of motion at the elbow joint. There was no radiographic evidence of arthritis in any of the specimens. In addition, no instability was observed during valgus and varus stress loading tests.

The upper limbs were separated from the torso at the level of the glenohumeral joint with a scalpel. Subcutaneous tissues and fascia were dissected to expose the insertion sites of the biceps brachii, brachialis, triceps brachii, supinator, and pronator teres. The insertion site of the tendon was sutured using the modified Krackow method with No. 5 Ethibond polyester suture (Ethicon, Somerville, NJ). A humeral intramedullary nail (Multiloc Humeral Nails; Synthes, West Chester, PA, US) was inserted using C-arm fluoroscopy to securely fix the specimen to a custom-designed testing apparatus. The humerus intramedullary nail was inserted to a depth that could protrude approximately 10 cm from the entry point of the humerus so that it could be fixed to the testing apparatus. In addition, two K-wires were inserted parallel to the shaft of the radius to fix the wireless inclinometer sensor (E2BOX, Hanam-si, Gyeonggi-do, South Korea). A careful incision was made in the anterior capsule of the elbow to insert a pressure sensor in the RCJ, avoiding damage to the collateral and annular ligaments. Afterwards, to preserve the physiological movement of the tendons as much as possible, the skin and soft tissue were closed in layers.

### Testing apparatus

**Figure 1:**
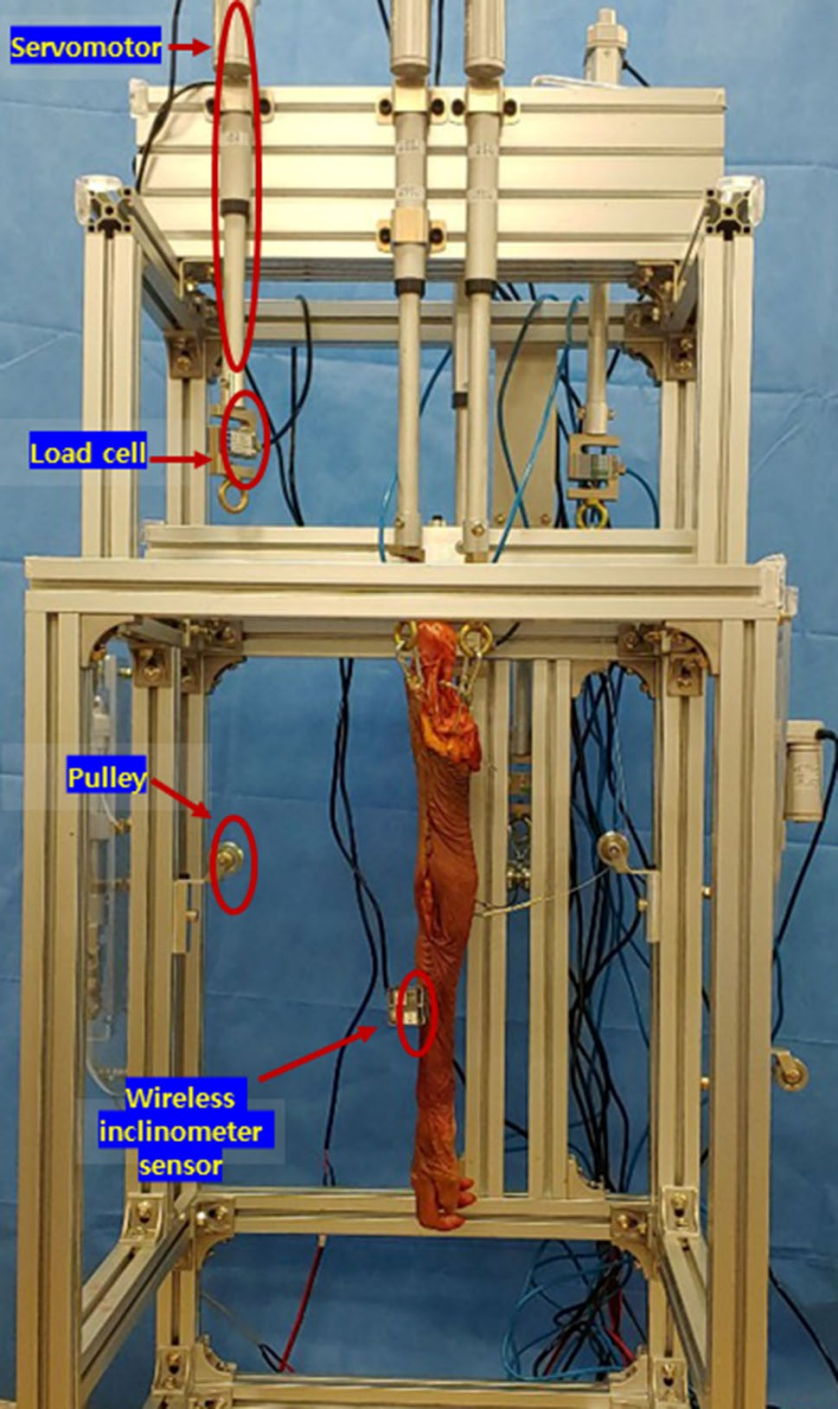
The testing apparatus is designed to provide independent load control for each tendon of the specimen so that flexion of the elbow joint and pronosupination of the forearm can be controlled.

**Figure 2:**
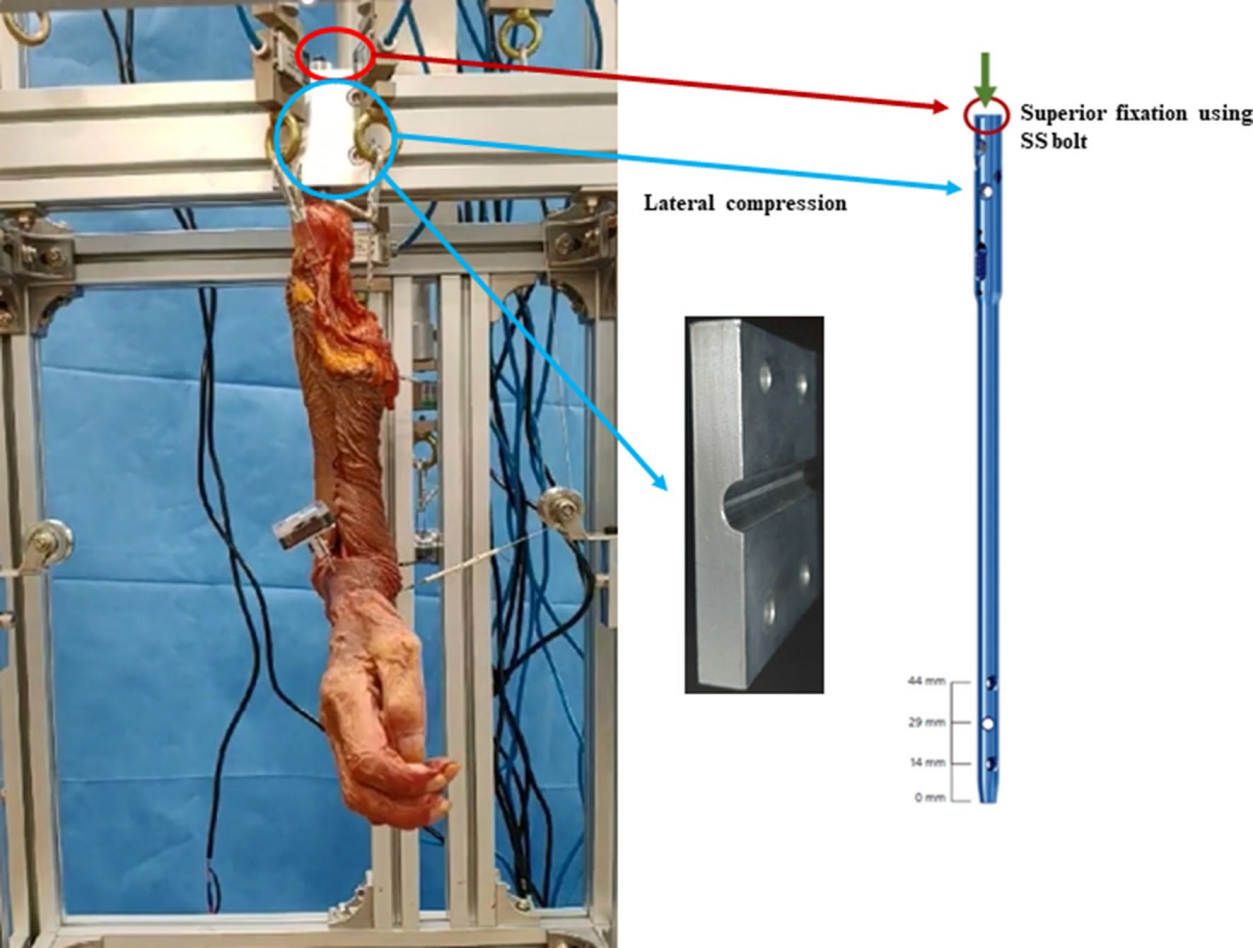
The specimen is primarily fixed to the testing apparatus using a bolt on the top end of a humeral intramedullary nail. The fixation is further secured by lateral compression of the protruding intramedullary nail. *SS* stainless steel.

The testing apparatus was designed to provide independent load control for each tendon of the specimen so that the motion of the elbow joint could be controlled (Fig. 1). Each specimen was primarily fixed to the testing apparatus using the bolt on the top end of the humeral intramedullary nail, and the fixation was further secured by lateral compression of the protruding intramedullary nail (Fig. 2). Five servomotors (JMC, Seoul, South Korea) were included on the testing apparatus, and wires were used to connect the suture in each tendon to the servomotors. The testing apparatus also included several pulleys, and the vector of the wire was controlled similarly to the physiological direction of each tendon by adjusting the position of each pulley. A load cell (CAS, Yangju-si, Gyeonggi-do, South Korea) was equipped on the wire connected to each servomotor so that the force loaded on the wire could be determined in real time. Each tendon was pulled using servomotors controlled by custom-designed software (Burnyoung, Daejeon, South Korea) and a servomotor controller (NTrex, Incheon, South Korea). The custom-designed software was developed not only to control the servomotor, but also to determine the force loaded on the wire through the load cell, and to determine the elbow flexion angle and forearm rotation angle with a wireless inclinometer sensor in real time.

A Tekscan sensor 6900 (Tekscan Inc., South Boston, MA, US) validated in prior studies was used for the pressure sensor^19,20^. The 6900 sensors were preconditioned and calibrated according to the manufacturer’s instructions and then inserted into the RCJ. Using the manufacturer’s software, the contact area, mean pressure, and peak pressure of the joint were measured in real time.

### Measurement and simulation of motion

**Figure 3:**
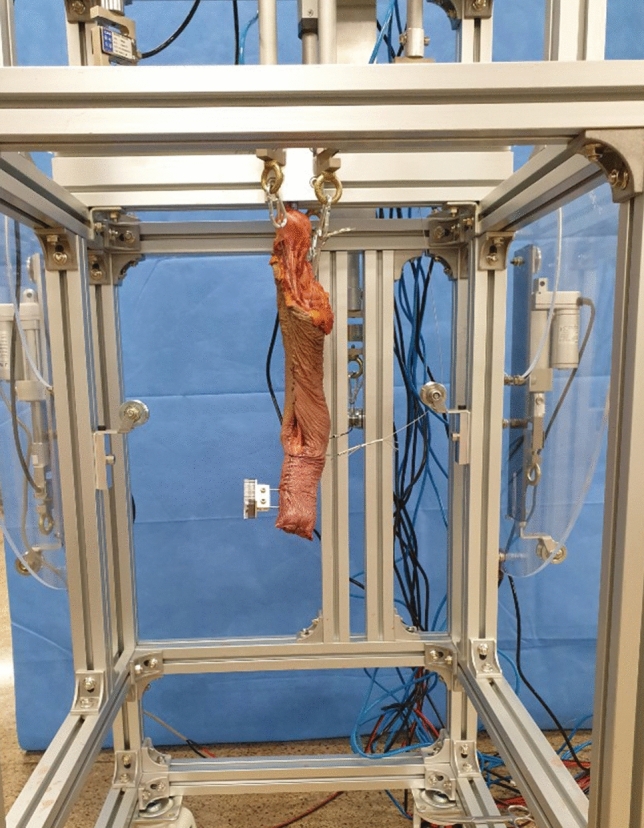
A transradial amputation was performed on each of the ten upper limb specimens. The amputations were performed 5 cm proximal to the radial styloid process.

The elbow flexion and forearm rotation angles of the specimens were determined using a wireless inclinometer sensor. The contact area, mean pressure, and peak pressure of the RCJ at various elbow flexion and forearm rotation angles were evaluated. Variables were measured at the neutral rotation of the forearm, 40° pronation, and 40° supination at each of the following elbow flexion angles: 0°, 45°, and 90°. The testing apparatus was stopped at each angle of elbow flexion and forearm rotation to measure the variables. At first, the variables were measured at an elbow flexion angle of 0° with the forearm rotation in neutral, 40° pronation, and 40° supination. Then, the experiments were repeated at angles of 45° and 90° of elbow flexion. The experiment was performed using normal upper limbs and then repeated at the same positions of the elbow after performing a transradial amputation. Transradial amputation was performed 5 cm proximal to the radial styloid process (Fig. 3). Skin incisions were designed with equal-length flaps along the volar and dorsal aspects of the forearm, and the skin and soft tissue were closed in layers after the osteotomy.

In the neutral rotation of the forearm at an elbow flexion angle of 0°, variables were measured after applying a 45 N load on the brachialis and triceps brachii^20^. During pronation of the forearm, the pronator teres was pulled at a speed of 5 mm/s, while a 20 N counterforce was loaded to the supinator. During supination of the forearm, the biceps brachii and supinator were pulled at a speed of 5 mm/s, while a 20 N counterforce was applied to the pronator teres^21^. In addition, the posture was maintained by controlling the counterforce on the triceps brachii so that the biceps brachii did not flex the elbow joint. During flexion of the elbow joint, the brachialis and biceps brachii were pulled at a speed of 5 mm/s, while a 20 N counterforce was applied to the triceps brachii. The loading ratios calculated from electromyographic activity and physiologic cross-sectional area reported in previous studies were 57% for the brachialis and 43% for the biceps brachii, showing similar loading ratios for the two flexors of the elbow joint^22–24^. At the elbow flexion angles of 45° and 90°, variables were measured at various positions while rotating the forearm by controlling the load of the pronator teres, biceps brachii, and supinator using the aforementioned protocol.

Based on the results of previous studies, in which microscopic damage to cartilage occurs when the peak pressure loaded on the articular cartilage exceeds 5 MPa^25,26^, we investigated whether the peak pressure of the RCJ in each normal and transradial amputated upper limb exceeded 5 MPa during elbow motion.

### Statistical analysis

The mean of three trials was calculated and used for statistical analyses. The comparisons of variables according to the elbow flexion angle and position of forearm rotation were performed using the Friedman test, and post-hoc analysis was performed using the Wilcoxon signed rank test. The Wilcoxon signed rank test was also used to compare normal and transradial amputated upper limbs. Statistical analyses were performed using SPSS version 18.0 (SPSS Inc., Chicago IL, US). A *P* value of less than 0.05 was considered statistically significant. In the post-hoc analysis, the significance level was corrected using Bonferroni’s method. Sample size was not calculated before the study given the absence of available comparable data in the literature; instead, a retrospective power analysis was performed using the G*power software package, version 3.1.9.2 (Heinrich Heine University Düsseldorf, Germany).

## Results

### Contact area

Changes in the contact area of the RCJ according to the elbow flexion and forearm rotation angles of the normal and transradial amputated upper limbs are presented in Table 1. At an elbow flexion angle of 0°, the contact area according to the position of forearm rotation was the largest at the pronation position in both groups (all *P* < 0.017, significant values were corrected by Bonferroni’s method) (Table 1). At an elbow flexion angle of 45°, the contact area was also the largest at the pronation position (all *P* < 0.017) (Table 1). However, at an elbow flexion angle of 90°, there were no significant differences in the contact area of the RCJ according to the position of forearm rotation in both groups.

**Table 1:** Contact area of the radiocapitellar joint at various degrees of elbow flexion and forearm rotation angles in normal and transradial amputated upper limbs.

Elbow flexion angles	Forearm rotation angles			P-value	Subgroup analysis*		
	Neutral	P40°	S40°		Neutral vs P40°	Neutral vs S40°	P40° vs S40°
**Normal upper limb**							
Flexion 0°	39 ± 5	54 ± 9	42 ± 9	**0.001**	**0.005**	0.097	**0.007**
Flexion 45°	49 ± 9	60 ± 10	47 ± 8	** < 0.001**	**0.005**	0.241	**0.007**
Flexion 90°	52 ± 5	53 ± 11	52 ± 10	0.922			
P value	**0.001**	00.061	0.132				
Subgroup analysis*							
0° vs 45°	**0.008**						
0° vs 90°	**0.005**						
45° vs 90°	0.152						
**Transradial amputated upper limb**							
Flexion 0°	38 ± 6	55 ± 8	43 ± 5	** < 0.001**	**0.005**	0.112	**0.005**
Flexion 45°	48 ± 7	58 ± 9	48 ± 5	**0.007**	**0.007**	0.959	**0.008**
Flexion 90°	52 ± 5	54 ± 6	50 ± 7	0.146			
P value	**0.006**	0.245	**0.006**				
Subgroup analysis*							
0° vs 45°	**0.014**		0.**009**				
0° vs 90°	**0.007**		0.**011**				
45° vs 90°	0.168		0.575				

P40°, 40° pronation; S40°, 40° supination.

Boldface indicates significance. Values are presented as mean ± SD (mm^2^).

*The significance level was corrected from 0.05 to 0.017 (5%/3) by Bonferroni's method.

When the contact area of the RCJ was compared between the normal and transradial amputated upper limbs at all positions, no significant difference was found (Table 2).

**Table 2:** Contact area of the radiocapitellar joint between normal and transradial amputated upper limbs.

	Normal upper limb	Transradial amputated upper limb	*P* value
**Elbow flexion 0°**			
Neutral rotation	39 ± 5	38 ± 6	0.506
40° pronation	54 ± 9	55 ± 8	0.645
40° supination	42 ± 9	43 ± 5	0.799
**Elbow flexion 45°**			
Neutral rotation	49 ± 9	48 ± 7	0.812
40° pronation	60 ± 10	58 ± 9	0.384
40° supination	47 ± 8	48 ± 5	0.112
**Elbow flexion 90°**			
Neutral rotation	52 ± 5	52 ± 5	0.931
40° pronation	53 ± 11	54 ± 6	0.906
40° supination	52 ± 10	50 ± 7	0.725

Values are presented as mean ± SD (mm^2^).

### Mean pressure

Changes in the mean pressure of the RCJ according to the elbow flexion and forearm rotation angles of the normal and transradial amputated upper limbs are presented in Table 3. At elbow flexion angles of 0° or 90° in normal upper limbs, there were no significant differences in the mean pressures of the RCJ according to the position of the forearm rotation (Table 3). However, at an elbow flexion angle of 45° in normal upper limbs, the mean pressure was the highest in the pronation position (all* P* < 0.017).

**Table 3:** Mean pressure of the radiocapitellar joint at various degrees of elbow flexion and forearm rotation angles in normal and transradial amputated upper limbs.

Elbow flexion angles	Forearm rotation angles			P-value	Subgroup analysis*		
	Neutral	P40°	S40°		Neutral vs P40°	Neutral vs S40°	P40° vs S40°
**Normal upper limb**							
Flexion 0°	354 ± 67	425 ± 108	383 ± 101	0.125			
Flexion 45°	872 ± 223	1052 ± 223	705 ± 89	** < 0.001**	**0.007**	0.022	**0.005**
Flexion 90°	805 ± 79	824 ± 101	759 ± 80	0.061			
P value	**0.001**	** < 0.001**	** < 0.001**				
Subgroup analysis*							
0° vs 45°	**0.005**	**0.005**	**0.005**				
0° vs 90°	**0.005**	**0.005**	**0.005**				
45° vs 90°	0.386	**0.005**	0.074				
**Transradial amputated upper limb**							
Flexion 0°	470 ± 72	559 ± 91	485 ± 72	** < 0.001**	**0.005**	0.086	**0.005**
Flexion 45°	1096 ± 197	1488 ± 284	860 ± 104	** < 0.001**	**0.005**	**0.008**	**0.005**
Flexion 90°	1007 ± 107	1056 ± 222	905 ± 100	0.407			
P value	** < 0.001**	** < 0.001**	**0.001**				
Subgroup analysis*							
0° vs 45°	**0.005**	**0.005**	**0.005**				
0° vs 90°	**0.005**	**0.005**	**0.005**				
45° vs 90°	0.028	**0.005**	0.333				

P40°, 40° pronation; S40°, 40° supination.

Boldface indicates significance. Values are presented as mean ± SD (kPa).

*The significance level was corrected from 0.05 to 0.017 (5%/3) by Bonferroni's method.

No significant difference in mean pressure according to the position of the forearm rotation was found at an elbow flexion angle of 90° in upper limbs after transradial amputation (Table 3). However, at elbow flexion angles of 0° or 45°, the mean pressure was highest in the pronation position in transradial amputated upper limbs (all P < 0.017) (Table 3).

In both groups, the mean pressure was higher at elbow flexion angles of 45° and 90° than at 0° in all forearm rotation positions (all *P* < 0.017) (Table 3). In addition, the mean pressure was significantly higher at 45° of elbow flexion than at 90° during pronation (all *P* < 0.017) (Table 3).

The mean pressure of the RCJ was significantly higher in the upper limbs after transradial amputation than in the normal upper limbs at all positions (all *P* < 0.05) (Table 4).

**Table 4:** Mean pressure of the radiocapitellar joint between normal and transradial amputated upper limbs.

	Normal upper limb	Transradial amputated upper limb	*P* value
**Elbow flexion 0°**			
Neutral rotation	354 ± 67	470 ± 72	**0.005**
40° pronation	425 ± 108	559 ± 91	**0.005**
40° supination	383 ± 101	485 ± 72	**0.007**
**Elbow flexion 45°**			
Neutral rotation	872 ± 223	1096 ± 197	**0.037**
40° pronation	1052 ± 223	1488 ± 284	**0.005**
40° supination	705 ± 89	860 ± 104	**0.022**
**Elbow flexion 90°**			
Neutral rotation	805 ± 79	1007 ± 107	**0.005**
40° pronation	824 ± 101	1056 ± 222	**0.005**
40° supination	759 ± 80	905 ± 100	**0.013**

Boldface indicates significance. Values are presented as mean ± SD (kPa).

### Peak pressure

**Figure 4:**
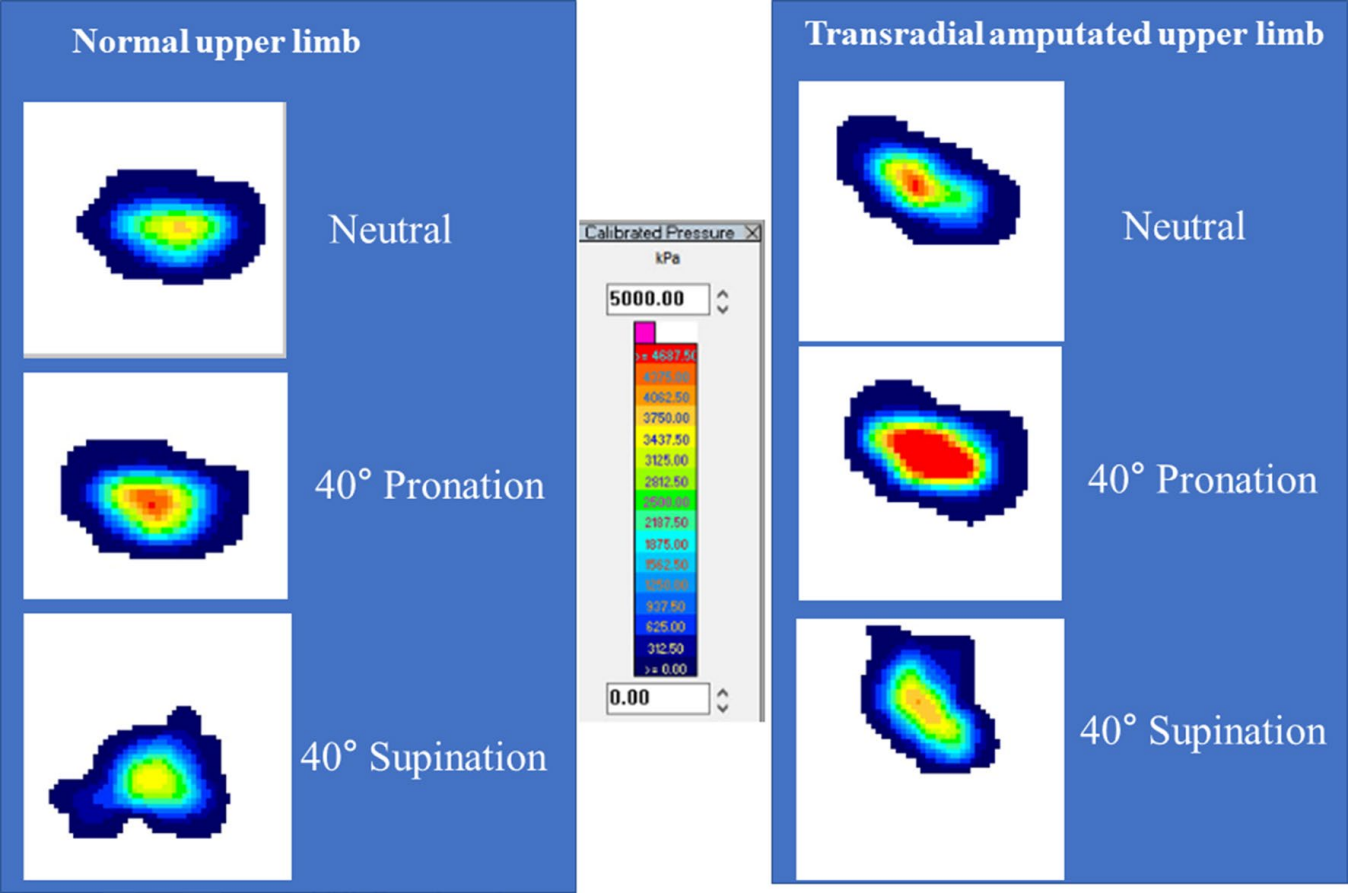
These colour maps show pressures at an elbow flexion angle of 45° in normal and transradial amputated upper limbs. Pressures were increased in the upper limbs after transradial amputation compared to the normal upper limbs. The increased pressures in the upper limbs after transradial amputation were most evident during pronation.

Similar to the mean pressure, the peak pressure was also significantly higher in the upper limbs after transradial amputation at all positions when compared to the normal upper limbs (all *P* < 0.05) (Table 5). The peak pressure was the highest during pronation at an elbow flexion angle of 45° in the upper limbs after transradial amputation. The peak pressure was not higher than 5 MPa in any position of the normal upper limb, however, the peak pressure was over 5 MPa for four of the ten specimens during pronation at the elbow flexion angle of 45° in the upper limbs after transradial amputation (Fig. 4).

**Table 5:** Peak pressure of the radiocapitellar joint between normal and transradial amputated upper limbs.

	Normal upper limb	Transradial amputated upper limb	*P* value
**Elbow flexion 0°**			
Neutral rotation	549 ± 105	848 ± 203	**0.005**
40° pronation	851 ± 215	1162 ± 206	**0.005**
40° supination	619 ± 143	914 ± 184	**0.005**
**Elbow flexion 45°**			
Neutral rotation	1907 ± 551	2810 ± 441	**0.008**
40° pronation	2468 ± 704	4213 ± 998	**0.005**
40° supination	1602 ± 177	1955 ± 216	**0.017**
**Elbow flexion 90°**			
Neutral rotation	1691 ± 165	2145 ± 283	**0.005**
40° pronation	1667 ± 209	2134 ± 450	**0.009**
40° supination	1588 ± 136	1862 ± 284	**0.047**

Boldface indicates significance. Values are presented as mean ± SD (kPa).

### Power analysis

The main question for the current study was whether changes in the biomechanics within the RCJ could increase the stress against the joint cartilage in transradial amputees. Therefore, the primary outcome measure of this study could be defined as the mean peak pressure loaded on the RCJ in each group. We selected the index position of the elbow flexion angle at 45° during pronation for the sample size calculation because the stress required to injure the joint cartilage was thought to be maximally increased at this posture. The mean peak pressure loaded on the RCJ in the two groups (normal and transradial amputated upper limbs) were 2468 ± 704 vs. 4213 ± 998 (kPa), respectively (Table 5). With the Wilcoxon signed rank test statistical model and an estimated correlation between the two groups of 0.44, the effect size was estimated to be 1.87. With an effect size of 1.87, a significance level of 0.05, and given a sample size of 10, the power of the current study was calculated to be 99.8%.

## Discussion

In the present study, the contact area, mean pressure, and peak pressure of the RCJ were analysed during elbow flexion and forearm rotation in normal and transradial amputated upper limbs. The contact area of the RCJ tended to increase during the pronation of the forearm, however, this tendency was not observed at the elbow flexion angle of 90°. No significant difference was found between the contact areas of the normal and transradial amputated upper limbs. However, the mean and peak pressure significantly increased in the upper limbs after transradial amputation compared to the normal upper limbs. Such pressure increase was evident during pronation at an elbow flexion angle of 45°.

With the advancement of bioengineering, the myoelectric prosthesis can be moved as intended by identifying the neural activity patterns from residual nerves^27,28^. In addition, natural forearm rotation can be restored through osseointegration of the prosthesis to the stump in transradial amputees^10^. Although forearm rotation is possible by the organic relationship among the elbow joint, forearm, and wrist joint^29^, the biomechanical changes in the elbow joint of transradial amputees are not well-known.

The mean and peak pressure of the RCJ significantly increased in the upper limbs after transradial amputation. This may be explained by forearm axial instability. The radial head, interosseous membrane, and triangular fibrocartilage complex (TFCC) are involved in forearm axial stability^18,30^. If significant damage occurs to these structures, axial instability may occur, ultimately leading to the proximal migration of the radius. In this study, amputation was performed 5 cm proximal to the radial styloid process, resulting in the loss of the DRUJ, TFCC, pronator quadratus, and DOB. The DOB attaches to the ulnar aspect of the radius at approximately 34 mm proximal to the radial styloid process^31^, and is known as a structure that provides secondary stability to the DRUJ^32,33^. Hwang et al.^34^ reported that the increase in the pressure and force of the RCJ is much higher after injuries to the interosseous membrane than injuries to the DRUJ. However, the authors mentioned that pressure in the RCJ increases only after injury to the DRUJ. In cases of transradial amputation, not only soft tissue stability but also bony stability is lost, which can lead to greater forearm axial instability. As a result, the pressure transmitted to the RCJ is increased.

A change in the ratio of force transmitted to the RCJ and ulnohumeral joints is another factor that may increase the pressure of the RCJ in the upper limbs after transradial amputation. When the ulnar variance is neutral in the normal upper limbs, 80% of the axial load is transmitted by the radiocarpal joint, and 20% is transmitted through the ulnocarpal joint^35^. Some portion of the force transferred to the radius is transmitted to the ulna through the interosseous membrane, resulting in the RCJ absorbing 60% of the axial load while 40% is transferred to the ulnohumeral joint^36,37^. Dissociation between the radius and the ulna occurs in transradial amputees causing normal force not transmitted through the interosseous membrane, which can cause an abnormally higher ratio of axial load on the RCJ than normal. The exact force ratio is unknown because the biomechanical indices of the ulnohumeral joint were not measured in this study. However, Hwang et al.^34^ reported that the axial load transmitted to the RCJ is 80% or higher when the interosseous membrane and DRUJ are injured.

The peak pressure on four of the ten upper limb specimens after transradial amputation was over 5 MPa. Previous studies reported that when a pressure greater than 5 MPa is repeatedly loaded onto the cartilage, damage to the cartilage begins at the microscopic level^25,26^. After radial head prosthetic replacement due to a radial head fracture, the peak pressure of the RCJ was reported to increase^19,38^. The erosion of cartilage and degenerative changes among patients who underwent a radial head replacement has been reported in several studies^39,40^. Similarly, the increased peak pressure transmitted to the RCJ in the upper limbs after transradial amputation may cause abnormal wear of the cartilage, resulting in radiocapitellar arthritis, elbow pain, and limitation of elbow motion. These findings can adversely affect the already limited function of a transradial amputee. Transradial amputation is frequently due to trauma in young people. Therefore, more attention should be paid to degenerative changes because the long-term prognosis is important in young patients. Thus, when developing a myoelectric prosthesis, it should be designed to minimise the axial instability of the forearm to reduce the pressure on the RCJ.

In the present study, there were no significant differences in the contact area of the RCJ between normal and transradial amputated upper limbs. The contact area has a limited range that can be increased compared to the wide range of the contact pressure. Since axial load was sufficiently transmitted to the RCJ in normal upper limbs, the RCJ contact area in the same respective angles of elbow flexion and forearm rotation in transradial amputated upper limbs remained the same, but had a significant increase in pressure. Another reason to consider for the lack of difference is the limitation of the Tekscan 6900 for accurately measuring the contact area of the RCJ. Although we attempted to fix the pressure sensor to the fovea of the radial head, the sensor still slightly wrinkled during the range of motion. In addition, the Tekscan 6900 did not cover the entire articulating aspect of the radial head^19,41^. The limitations of the sensor should be considered when interpreting the results of the contact area.

An in vivo three-dimensional elbow biomechanical study reported that during forearm pronation, the ulna shows a valgus rotation and the radial head is translated anteriorly and proximally^42^. Palmer and Werner also reported that the radius moves proximally during forearm pronation^35^. Other studies reported that the RCJ is tighter, and greater force is transmitted in pronation than in supination^37,43^. For these reasons, the contact area of the RCJ is larger and the mean pressure is higher in pronation. In the present study, the contact area tended to increase during pronation compared to the neutral position or supination, however, this tendency was not observed at the elbow flexion angle of 90°. When the elbow joint is flexed, the contact area of the RCJ moves anteriorly^44^, and the contact area migrates further anteriorly during pronation^42^. Such excessive anterior movement of the radial head may reduce the contact area^44^. Although it was not significant, the contact area at the elbow flexion angle of 90° was slightly decreased compared to the elbow flexion angle of 45° during pronation. As a result, there was no significant increase in the contact area during pronation at the elbow flexion angle of 90° compared to other rotational positions.

The mean pressure was higher at elbow flexion angles of 45° and 90° compared to 0°. These results could be caused by lack of muscle tensioning at an elbow flexion angle of 0° in cadaveric specimens. A force of 45 N was loaded to the brachialis and triceps at an elbow flexion angle of 0° because there was no pressure transmitted to the RCJ if no force was applied to any tendon in the cadaveric specimens^20^. Although 45 N of tension was applied to the brachialis and triceps^20^, the force transmitted to the RCJ was lacking compared to the force applied during elbow flexion. During elbow flexion, a 20 N counterforce of the triceps was applied and the tendons of the biceps and brachialis were pulled at a speed of 5 mm/s. Although the exact degree of force was not measured due to continuous change in real time, it was confirmed that a force of 45 N or more was applied to the tendons of the biceps and brachialis during elbow flexion. In particular, the initial force loaded on the tendon at the beginning of flexion was high but decreased near the end of flexion. Therefore, the mean pressure at the elbow flexion angle of 45° was higher than that at 90°. An increase in the mean pressure at the mid-flexion angle has been observed in previous studies^19,37,41^. The force loaded on the biceps brachii at the elbow flexion angle of 45° was relatively higher than at other angles, and a large amount of force was loaded on the pronator teres during pronation of the supinated forearm by the biceps brachii. As a result, it was determined that the mean and peak pressure increased the most during pronation at the elbow flexion angle of 45°.

The strengths of this study are as follows: First, this is the first study to analyse changes in biomechanical indices in the RCJ in upper limbs after transradial amputation. In the past, the functional aspect of transradial amputees was neglected, however, interest in this topic has increased due to the possibility of reproducing various movements with the recent development of the myoelectric prosthesis^6,10^. Accordingly, it is necessary to analyse the influence on the adjacent joint to the stump, and the results may provide important information for the development of the myoelectric prosthesis. Second, although it was conducted on cadaver specimens, biomechanical indices within the RCJ at various positions were measured by reproducing the elbow flexion and forearm rotation similar to in vivo motion. In vivo biomechanical studies have some weaknesses that cannot directly measure the pressure within the joint^42,44^. To the best of our knowledge, there has been no cadaveric study that measures biomechanical indices of the RCJ after reproducing the elbow joint flexion and forearm rotation by applying force to the tendons.

This study has several limitations. First because this study used cadaveric specimens, the degree of tension of the soft tissues was not similar to that which would occur in vivo. A force of 45 N was loaded to the brachialis and triceps brachii at an elbow flexion angle of 0°, but it was significantly less than the degree of soft tissue tension caused by elbow flexion, and a low mean pressure was measured at the elbow flexion angle of 0°. Different results may be obtained depending on the degree of muscle tension in vivo. Second, the pronator quadratus was not considered during pronation, as the pronator quadratus is obliterated in transradial amputees. However, the contribution of the pronator quadratus for pronation is smaller compared to the pronator teres^24^, and the pulling of the pronator teres alone can produce pronation similar to the simultaneous pulling of the pronator quadratus and pronator teres^15^. Third, transradial amputation was not performed at various levels. A previous study reported that if the length of the forearm is 18 cm or longer, 80% of forearm rotation is preserved^15^, and we performed amputation at 5 cm proximal to the radial styloid process. If the amputation is more proximal, the active pronosupination arc is greatly reduced, thus, a rotational function should be added to the prosthesis. Fourth, although the contact area and pressure can be directly measured using the Tekscan sensor, the sensor can be slightly wrinkled during the range of motion when it is used on non-planar joint surfaces, which should be considered when interpreting the results, especially regarding the contact area. In addition, the Tekscan sensor 6900 used in this study was not able to include the rim of the radial head; thus, the biomechanics of the radial head rim could not be assessed^19^. However, the 6900 pressure transducer has been used in several studies to investigate the contact area and pressure in the RCJ^19,20,41^. Finally, as transradial amputation occurs most frequently in patients at a young age^3^, the advanced mean age of the cadavers may lead to differences in the results.

The mean and peak pressures of the RCJ were significantly higher in the transradial amputations when compared to the normal upper limbs. These increases were especially evident during mid-flexion and pronation. These results could cause cartilage erosion and arthritis in the RCJ of transradial amputees. Thus, methods to reduce the axial instability of the forearm should be considered when a myoelectric prosthesis is developed.

## Data Availability

The data used to support the findings of this study are available from the corresponding author upon request.
